# PacBio full-length 16S rRNA gene sequencing processed with Emu and GTDB provides the highest taxonomic resolution for rumen bacteriome profiling

**DOI:** 10.1093/ismeco/ycag148

**Published:** 2026-05-29

**Authors:** Hanbeen Kim, Mi Zhou, Limei Lin, Wen Zhu, Tim A McAllister, Le Luo Guan

**Affiliations:** Faculty of Land and Food Systems, The University of British Columbia, Vancouver, BC V6T 1Z4, Canada; Faculty of Land and Food Systems, The University of British Columbia, Vancouver, BC V6T 1Z4, Canada; Faculty of Land and Food Systems, The University of British Columbia, Vancouver, BC V6T 1Z4, Canada; Faculty of Land and Food Systems, The University of British Columbia, Vancouver, BC V6T 1Z4, Canada; College of Animal Science and Technology, Anhui Agricultural University, Hefei, Anhui 230036, PR China; Agriculture and Agri-Food Canada, Lethbridge Research Centre, Lethbridge, AB T1J 4B1, Canada; Faculty of Land and Food Systems, The University of British Columbia, Vancouver, BC V6T 1Z4, Canada

**Keywords:** rumen, bacteriome, full-length 16S rRNA gene sequencing, taxonomic resolution, reference database limitation, Pacific biosciences, Oxford nanopore technologies, Emu, GTDB

## Abstract

Although full-length 16S rRNA gene sequencing has substantially improved taxonomic resolution compared to short-read approaches, a high proportion of unclassified taxa are reported in rumen microbiome studies. This limitation is largely driven by platform-specific analytical workflows and the insufficient representation of rumen-associated lineages in commonly used reference databases. Here, we identified the optimal combination of sequencing platform, analytical workflow, and reference database to improve rumen bacteriome classification. We analyzed short-read and full-length 16S rRNA gene sequences from rumen samples collected from two beef cattle populations. Short-read sequences were generated using Illumina NextSeq2000 and processed with QIIME2. Full-length sequences were generated using PacBio Revio (PacBio-16S) and Nanopore MinION (ONT-16S); PacBio-16S data were analyzed using QIIME2 and Emu, while Nanopore data were analyzed using EPI2ME and Emu. Five reference databases were evaluated across all analytical approaches: SILVA 138.2, SILVA 138.2 with Hungate1000 collection, NCBI, Greengenes2, and GTDB. The comparisons showed that PacBio-16S (Emu) achieved the highest proportion of classified reads among all platform-specific workflows, while GTDB consistently produced the highest number of non-redundant classified taxa. *Prevotella*, a dominant rumen genus, was abundant in Illumina and PacBio-16S datasets but was underrepresented in ONT-16S workflows. Species-level analyses further demonstrated that PacBio-16S (Emu) reliably provided more consistent and high-resolution identification of *Prevotella* species under GTDB across two beef populations. Overall, our results demonstrate that sequencing platform, workflow choice, and database selection strongly influence rumen bacteriome profiles. We recommend PacBio-16S (Emu) under GTDB as the most reliable workflow for achieving high-resolution taxonomic classification of rumen bacteriome.

## Main body

Third-generation sequencing platforms such as Pacific Biosciences (PacBio) and Oxford Nanopore (ONT) have demonstrated improved taxonomic resolutions and reduced ambiguities compared to short-read methods by capturing the full-length 16S rRNA gene sequences [1]. However, despite these technical advantages, several studies using full-length 16S rRNA gene sequencing to profile the rumen microbiome have reported a substantial proportion of unclassified bacterial reads at the species level [2–4]. This outcome could reflect the limited representation of rumen-associated lineages in conventional reference databases [5]. SILVA remains one of the most widely used reference databases for 16S rRNA gene-based studies, but it does not readily support species-level classification [6]. Although the incorporation of rumen-specific genomes, such as those in the Hungate1000 collection [7] has improved classification accuracy [5], representation of rumen bacteria is still limited. Alternative databases including NCBI, Greengenes 2 (GG2), or GTDB, incorporate environmentally metagenome-assembled genomes and have progressively expanded taxonomic coverage beyond traditional reference collections, enabling improved classification of full-length 16S rRNA gene amplicon sequencing [8]. However, it is unclear how these reference databases could improve the taxonomic classification of rumen bacteria. In addition to the reference databases, processing bioinformatic pipelines also presents a challenge. PacBio full-length 16S rRNA gene (PacBio-16S) reads are commonly processed using the DADA2 denoise-ccs and Naïve Bayes classifier within QIIME2 [9], while for ONT full-length 16S rRNA gene (ONT-16S) sequences, EPI2ME (wf-16s) platform is often used for taxonomic assignment. Additionally, Emu applies an expectation–maximization algorithm to reduce false positives and negatives arising from both ONT and PacBio reads [10]. In this study, we conducted a comparative analysis of sequencing platforms, bioinformatic pipelines, and reference databases to identify best practices for taxonomic profiling of the rumen bacteriome.

**Figure 1:**
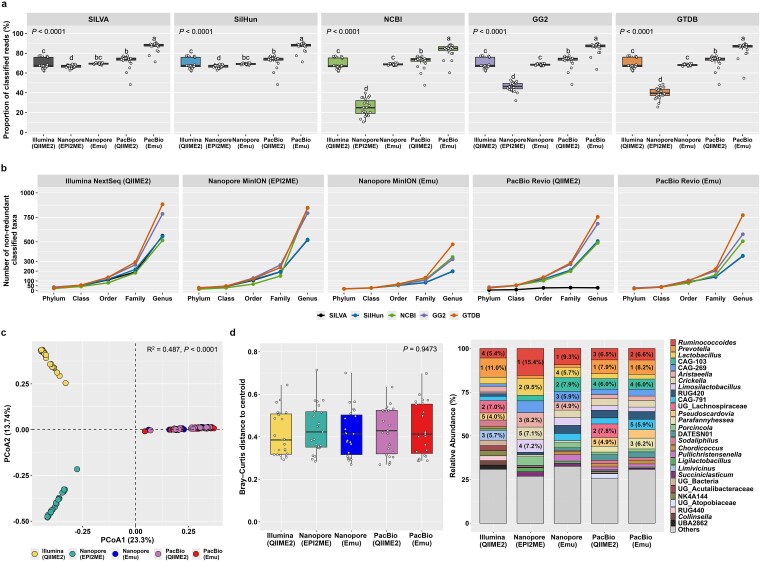
**Impact of sequencing platform and reference database on taxonomic resolution of the rumen bacteriome from 23 Kinsella composite hybrid steers.** (a) Proportion of classified reads (%) across sequencing platforms using different reference databases. Different letters indicate significant differences among platforms within each database. (b) Number of non-redundant classified taxa across taxonomic ranks for each sequencing platform and reference database. Non-redundant classified taxa were defined as the number of unique taxonomic labels at each rank after excluding unassigned taxa. (c) Principal coordinates analysis based on bray–Curtis dissimilarity at the genus level under GTDB classification. (d) Beta diversity dispersion measured as the distance to centroid based on bray–Curtis dissimilarity. (e) Taxonomic distribution of major genera across sequencing platforms under GTDB classification. Major genera were defined as those with a prevalence ≥50% of samples within each sequencing platform and a relative abundance ≥2.0% in at least one platform. Only these major genera are shown. “Others” represents remaining taxa not included among the major genera. Numbers within bars indicate rank and relative abundance of the top five genera within each platform.

**Figure 2:**
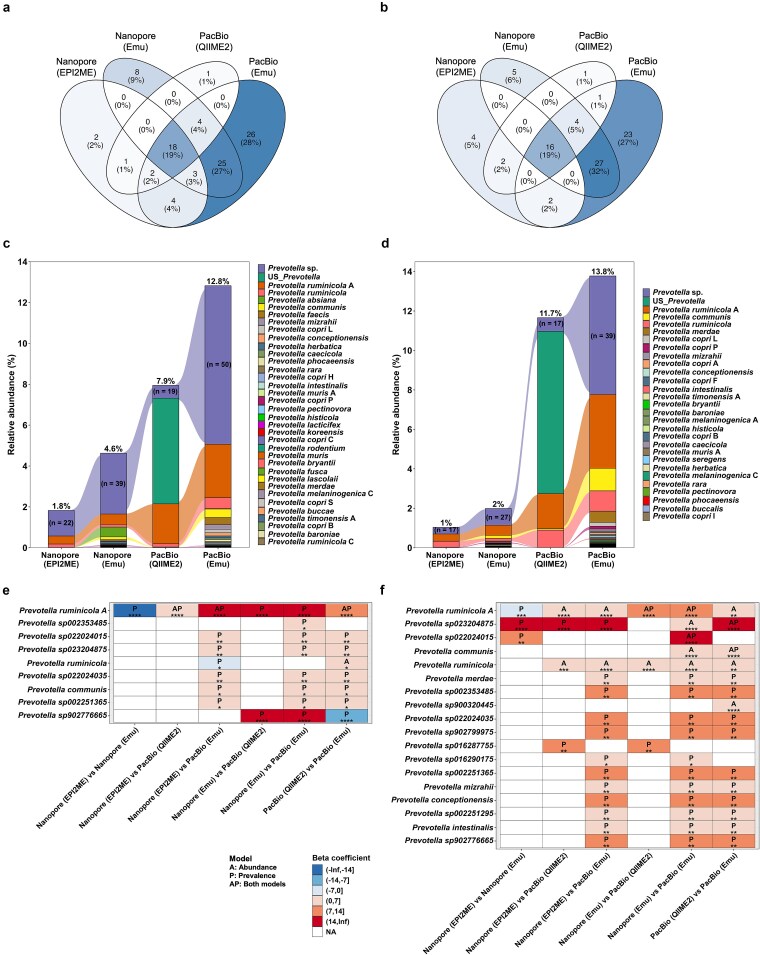
**Comparison of *Prevotella* species across sequencing platforms based on the GTDB (release 10-RS226) database.** (a–b) Venn diagrams showing the number and proportion (%) of shared and unique *Prevotella* species identified across sequencing platforms in (a) 23 Kinsella composite hybrid steers and (b) 24 Angus bulls. (c–d) relative abundance profiles of *Prevotella* species across sequencing platforms in (c) 23 Kinsella composite hybrid steers and (d) 24 Angus bulls. Bars represent cumulative relative abundance, with individual colors indicating species-level contributions. (e–f) pairwise differential abundance and prevalence analysis of *Prevotella* species across sequencing platforms based on MaAsLin3. Only *Prevotella* species with a prevalence ≥50% of samples within each sequencing platform and a relative abundance ≥0.05% in at least one platform were included in the analysis. Significant associations (q < 0.05) are visualized. Tiles are colored according to beta coefficients, and labels indicate the dominant model type: A, abundance; P, prevalence; AP, both models with concordant direction. When abundance and prevalence models showed opposite directions, the model with the larger absolute beta coefficient was selected. US_*Prevotella*, unclassified *Prevotella* species.

The sequence data of 23 rumen samples collected from Kinsella Composite hybrid (KC) steers [11] were generated and processed using short-read (Illumina NextSeq2000 with QIIME2) and full-length 16S rRNA gene sequencing (PacBio Revio with QIIME2 and Emu; and ONT MinION with EPI2ME and Emu). Species-level resolution was further evaluated using full-length 16S rRNA datasets from both KC steers (*n* = 23) [11] and Angus bulls (*n* = 24) [12], enabling assessment of the consistency of classification patterns across independent rumen populations. Taxonomic profiling was then performed using five reference databases–SILVA 138.2 (SILVA), SILVA 138.2 supplemented with the Hungate1000 collection (SilHun), NCBI 16S RefSeq (NCBI, downloaded 2025.06.17), GG2 (Release 2024.9), and GTDB (Release 10-RS226, v226). The performance of each sequencing platform and database was evaluated based on the average proportion of classified reads, diversity metrics, cross-method concordance, and differential abundance analyses. Detailed animal information, experimental methods, and bioinformatic workflows are provided in the Supplementary Information and Fig. S1. Although the ONT-16S exhibited a relatively lower proportion of bases with Q-scores $\ge$30 compared to the other platforms, all three sequencing platforms exhibited high read-level quality metrics (Tables S1–S3). When comparing classified read proportions, PacBio-16S (Emu) data yielded a significantly higher proportion of classified reads than other approaches across all reference databases (Fig. 1a; *P* < .0001). Due to limitations in species-level identification for short-read amplicon data [1], comparisons among sequencing platforms were conducted at the genus level. Rarefaction curves based on the observed genera indicated adequate sequencing depth across all platforms (Fig. S2). Across databases, GTDB consistently yielded the highest number of non-redundant classified taxa from phylum to genus levels (Fig. 1b), as well as the highest number of observed genera, followed by GG2 (Fig. S3; *P* < .0001). However, a greater number of detected genera does not necessarily indicate improved taxonomic accuracy or resolution. Although Illumina-QIIME2 with GTDB detected a greater number of genera, this may partly reflect inflated diversity estimates associated with short-read amplicon sequencing. Previous study has reported that short-read regions may also overestimate bacterial diversity compared with full-length 16S rRNA gene sequencing [13]. In contrast, PacBio full-length sequencing combined with Emu provided more consistent taxonomic profiles across databases and may therefore better reflect biologically robust genus-level classification. The superior performance of GTDB is attributable to its expanded genomic representation [14]. However, caution is warranted when interpreting these results, as the inclusion of MAGs may introduce limitations, including genome incompleteness and potential contamination, which may affect the reliability of taxonomic assignments. In the beta diversity, Illumina-derived profiles were largely separated from long-read sequencing platforms across most databases, except SILVA where separation was less distinct (Fig. S4, *P* < .0001). Long-read workflows showed relatively tighter clustering, particularly under SilHun, NCBI, and GTDB classifications (Fig. 1c and Fig. S4). *Prevotella* is widely recognized as a dominant and functionally important genus in the rumen [15, 16]. Under GTDB classification, *Prevotella* was the most predominant genus in Illumina (QIIME2) and PacBio-16S (QIIME2 and Emu), whereas *Ruminococcoides* was dominant in both EPI2ME and Emu workflows for ONT-16S (Fig. 1d). *Prevotella* was also detected among the major genera under GG2 and SilHun classifications, although with lower relative abundance. In contrast, it was not identified as a dominant genus under SILVA and NCBI classifications, indicating substantial database-dependent variation in taxonomic assignments (Fig. S5). Based on its superior performance in taxonomic coverage and cross-platform consistency, GTDB was selected for subsequent species-level analyses. PacBio-16S (Emu) enabled the capture of a higher effective number of species and a greater number of major bacterial species, with consistent patterns observed across both datasets (Fig. S6). Spearman correlation of effective Shannon diversity was used to assess concordance in alpha diversity estimates across sequencing platforms and analytical workflows. The highest correlation was observed between PacBio-16S workflows (QIIME2 and Emu), while Nanopore workflows (EPI2ME and Emu) also showed strong agreement (Fig. S7). These results suggest that the choice of sequencing platform has a stronger impact on overall community structure than the choice of analysis pipeline. Interestingly, Emu-based analyses exhibited relatively strong correlations between PacBio and Nanopore datasets, indicating the analysis pipeline can partially mitigate platform-specific differences. Although the distribution of major genera varied between KC steers and Angus bulls, *Prevotella* consistently remained the most abundant genus in both PacBio-16S workflows under GTDB, whereas *Ruminococcoides* and *Aristaeella* were predominant in ONT-16S workflows in KC steers and Angus bulls, respectively (Fig. S8A). In addition, differential abundance analysis revealed that *Prevotella* was the only genus that consistently exhibited concordant differential patterns across both datasets, remaining significantly enriched in PacBio-16S relative to ONT-16S regardless of analytical workflow (Fig. S8B). We further investigated species-level variation within the *Prevotella* to assess how sequencing platform and analytical workflow influence taxonomic resolution. Notably, PacBio-16S (Emu) consistently identified a greater number of *Prevotella* species (KC steers, *n* = 82 and Angus bulls *n* = 73) and captured a broader distribution of species-level abundances (KC steers = 12.8% and Angus bulls = 13.8%) compared to other workflows (Fig. 2a–d and Tables S4–S5). In addition, *Prevotella* species showed significant and concordant enrichment patterns in PacBio-16S relative to ONT-16S, with PacBio (Emu) providing the most consistent and robust signals across comparisons (Fig. 2e–f). Consistent with our findings, several studies have reported the potential underestimation of *Prevotella* in ONT-16S data [17–19]. Several technical factors may contribute to the observed underrepresentation of *Prevotella* species in ONT-16S. In particular, differences in PCR conditions (e.g. types of Taq polymerase, PCR cycles) and primer sequence (e.g. degenerate primers) could influence amplification biases across taxa [20]. As the primer information for the ONT 16S Barcoding Kit is not publicly disclosed, their taxonomic coverage cannot be directly evaluated, representing a limitation of this study. Future studies are needed to evaluate how PCR conditions and primer sequences in ONT-16S workflows contribute to biases in species-level resolution. Another limitation of this study is the absence of mock community validation. Although mock communities are useful for benchmarking accuracy, standardized rumen-specific mock communities remain limited, and available standards do not fully represent rumen microbial complexity. In addition, this study was based on two beef cattle cohorts, which may limit the generalizability of the findings to other ruminant systems. In conclusion, our findings demonstrate that PacBio (Emu) classified using GTDB provides improved species-level resolution and more consistent taxonomic profiling, particularly for ecologically important rumen bacterial taxa such as *Prevotella*.

## Supplementary Material

Supplementary_material_ycag148

## Data Availability

The 16S rRNA gene sequencing data are accessible under the NCBI BioProject (PRJNA1378457).
