# Supplementary material for: PacBio full-length 16S rRNA gene sequencing processed with Emu and GTDB provides the highest taxonomic resolution for rumen bacteriome profiling
Source: ISME Commun. 2026 May 29;6(1):ycag148. doi: 10.1093/ismeco/ycag148 (PMC13289738; doi:10.1093/ismeco/ycag148)
Supplement: Supplementary_material_ycag148 [file supplementary_material_ycag148.zip › 260526_Supplemental_Information.docx]

**Supplementary information**

**Method**

**Animal experiments, rumen sampling, and DNA extraction**

For the first comparative analysis of short-read and full-length 16S rRNA gene sequencing, we used 23 rumen samples collected from Kinsella composite hybrid raised under feedlot conditions at the Roy Berg Kinsella Research Ranch, University of Alberta. Detailed information on animal management, diet composition, and sample collection has been described previously [1]. Briefly, rumen digesta samples were collected at slaughter, snap-frozen in liquid nitrogen, and stored at $-$80°C unitil DNA extraction. To evaluate the reproducibility and robustness of Oxford Nanopore (ONT-16S) and Pacific Biosciences (PacBio-16S) full-length 16S rRNA gene sequencing workflows, we used 24 rumen samples from Angus bulls raised at the same facility. Detailed information on animal management, diet composition, and sample collection has been reported previously [2]. Briefly, approximately 50 mL of rumen contents (fluid and digesta) was collected from each animal using oro-gastric tubing as previously described [3], immediately frozen on dry ice, and stored at $-$80°C unitil DNA extraction. Total genomic DNA was extracted from each rumen sample using the repeated bead-beating plus column (RBB+C) method [4]. DNA quality and concentration were assessed using a NanoDrop ND-1000 Spectrophotometer (Thermo Fisher Scientific Inc., Wilmington, DE, USA).

**Short read amplicon sequencing**

16S rRNA gene amplicon sequencing was carried out at the Biofactorial Life Sciences Centre (University of British Columbia, Vancouver, Canada). Dual-indexed amplicon libraries were generated using a one-step PCR amplification strategy in a total reaction volume of 10 μL. PCR setup and liquid handling were automated on a Labcyte Access Workstation (Beckman Coulter). Each reaction contained 1 ng of input DNA and Quanta repliQa HiFi ToughMix (QuantBio), together with complete fusion primers incorporating Illumina Nextera adapters, dual indices, and region-specific primer pair 27F/338R targeting the V1-V2 region of the 16S rRNA gene (Ba27F: AGAGTTTGATCMTGGCTCAG and Ba338R: TGCTGCCTCCCGTAGGAGT) [5-7], as previously described [8]. The selection of the V1–V2 region was based on both literature support and technical considerations. In plant-associated bacterial genera, which are relevant to the rumen environment as it is enriched in plant-degrading taxa, Hrovat et al [7] reported that the V1–V3 region generally provides higher taxonomic resolution than the widely used V3–V4 region. However, previous studies have also shown that longer amplicons such as V1–V3 can introduce increased bias in community composition estimates compared to shorter regions such as V1–V2 [9]. In addition, longer amplicons can lead to reduced read merging efficiency and lower retention of filtered reads in short-read workflows [10]. Therefore, the V1–V2 region was selected as a practical compromise between taxonomic resolution, bias, and data retention. PCR amplification was performed with an initial denaturation at 98 °C for 120 s; 30 cycles of denaturation at 98 °C for 10 s, annealing at 50 °C for 5 s, and extension at 68 °C for 1 s; followed by a final hold at 6 °C. Amplified products were quantified using a PicoGreen fluorescence assay (Quant-iT™ PicoGreen™ dsDNA Assay Kit, Thermo Fisher Scientific). Equal amounts (2 ng) of each amplicon were pooled and purified using the AMPure XP PCR cleanup protocol (Beckman Coulter). The final pooled library was quantified again using PicoGreen and sequenced on an Illumina NextSeq2000 platform using a NextSeq P1 600-cycle XLEAP kit, following the manufacturer’s recommendations. A 25% PhiX spike-in was included to increase sequence diversity.

**PacBio Full-length 16S rRNA gene sequencing**

Full-length 16S rRNA gene amplification, SMRTbell library preparation, and sequencing were performed at Canada’s Michael Smith Genome Sciences Centre (Vancouver, Canada). Bacterial DNA samples were quantified using the Qubit 1× dsDNA High Sensitivity assay (Thermo Fisher Scientific) and normalized to 1 ng/µL in EB buffer (Qiagen). Fragment size profiles of input DNA were assessed using the Femto Pulse system (Agilent Technologies) to ensure DNA integrity prior to amplification. The universal primer-set were used to amplify the full-length 16S rRNA gene (27F: AGRGTTYGATYMTGGCTCAG and 1492R: RGYTACCTTGTTACGACTT) and to incorporate sample-specific indices for multiplexing. Four–base pair flanking buffer sequences were added to both ends of the primers to facilitate downstream library preparation. PCR amplification was performed in 25 µL reactions containing 5 µL PCR-grade water, 12.5 µL Q5 High-Fidelity 2× Master Mix (New England Biolabs), 6 µL primer mix (10 µM each of forward and reverse primers), and 2 µL template DNA (2 ng input). PCR cycling conditions consisted of an initial denaturation at 95 °C for 3 min, followed by 20 cycles of 95 °C for 30 s, 57 °C for 30 s, and 72 °C for 45 s, with a final hold at 4 °C. Following amplification, PCR products were purified using a 1× SPRI bead cleanup (PCRClean DX paramagnetic beads; Aline Biosciences LLC). Purified amplicons were quantified using the Qubit 1× dsDNA HS assay, and size distributions were confirmed for representative samples using the Agilent Bioanalyzer High Sensitivity DNA chip (Agilent Technologies). Samples were pooled in equimolar amounts, and the pooled library was again assessed on the Bioanalyzer. SMRTbell libraries were constructed from 500 ng of pooled amplicon DNA using the SMRTbell® Preparation Kit 3.0 (Pacific Biosciences), following the manufacturer’s protocol for multiplexed metagenome amplicon libraries. Briefly, DNA damage repair and end repair were performed, followed by ligation of SMRTbell index adapters and a 1× SPRI bead cleanup. Nuclease treatment was used to remove unligated DNA, followed by SMRTbell bead cleanup (Pacific Biosciences). The indexed SMRTbell libraries were processed using the Revio Polymerase Kit (Pacific Biosciences) according to the manufacturer’s instructions to generate final sequencing-ready libraries. Sequencing was performed on the PacBio Revio system at a target loading concentration of 300 pM, following standard manufacturer-recommended conditions.

**Oxford Nanopore Full-length 16S rRNA gene sequencing**

Full-length 16S rRNA gene amplification, library preparation, and sequencing were performed using the Oxford Nanopore MinION Mk1D platform with the 16S Barcoding Kit v14 (SQK-16S114-24; Oxford Nanopore Technologies, ONT), following the manufacturer’s protocol. Genomic DNA quality was assessed using Nanodrop, and DNA concentration was quantified using a Qubit fluorometer (Thermo Fisher Scientific). For each sample, 10 ng of genomic DNA was used as input for barcoded PCR amplification. Barcoded full-length 16S rRNA gene amplification (primer sequences not disclosed by manufacturer) was conducted using LongAmp® Hot Start Taq 2× Master Mix (New England Biolabs). Each 50 µL PCR reaction consisted of 10 ng genomic DNA, 25 µL LongAmp Hot Start Taq Master Mix, 10 µL of a unique 16S barcode primer from the kit, and nuclease-free water. PCR was performed with an initial denaturation at 95 °C for 60 s; 25 cycles of denaturation at 95 °C for 20 s, annealing at 55 °C for 30 s, and extension at 65 °C for 2 min; followed by a final extension at 65 °C for 5 min and hold at 4 °C. PCR reactions were terminated by addition of EDTA, and barcoded amplicons were quantified using a Qubit assay. Samples were pooled in equimolar ratios and purified using AMPure XP beads at a 0.6× bead-to-sample ratio. Following bead cleanup, the pooled library was eluted in elution buffer, quantified, and 50 fmol of DNA was used for adapter attachment. Rapid adapters were ligated according to the kit instructions, and the prepared library was kept on ice prior to loading. Flow cell quality was assessed using MinKNOW software, and only flow cells with more than 800 active pores were used for sequencing. The flow cell was primed and loaded according to ONT guidelines, with the sequencing library loaded using library beads. Sequencing was performed on the MinION Mk1D device for up to 72 h. Real-time base calling was disabled during the run, and raw signal data were collected for downstream post-run base calling using Dorado on high-performance computing resources.

**Bioinformatic analysis**

Bioinformatic processing was performed separately for each sequencing platform and analytical workflow, as summarized in Supplementary Figure 1. Where possible, consistent quality thresholds and taxonomic filtering criteria were applied across platforms.

*1. Illumina NextSeq 2000 (short-read 16S amplicon sequencing)*

Raw paired-end Illumina reads were initially subjected to quality assessment using a custom Python script. Adapter and primer sequences were removed using the cutadapt plugin implemented in QIIME2 (version 2025.7). Quality filtering and denoising were performed using DADA2 within QIIME2, applying default parameters optimized for paired-end data. Reads with an average Phred quality score below Q20 were excluded. Amplicon sequence variants (ASVs) generated by DADA2 were used for downstream taxonomic assignment using the classify-sklearn Naïve Bayes classifier in QIIME2 with the corresponding reference databases.

*2. Oxford Nanopore MinION*

For ONT data, raw signal files were base called using the SUP model in Dorado (version 1.0.2). Demultiplexing was also performed using Dorado. Following base calling, read-level quality metrics were assessed using a custom Python script. Reads were filtered by quality and length using chopper (v0.12.0b), retaining reads with an average Phred quality score ≥ Q20 and lengths between 1,200 and 1,600 bp, corresponding to full-length 16S rRNA genes. Post-filtering quality checks were conducted to confirm read retention and length distributions.

Two analytical workflows were applied to ONT data. For the EPI2ME workflow, filtered reads were directly subjected to taxonomic assignment using the EPI2ME wf-16s pipeline (v1.6.0). In parallel, filtered reads were analyzed using Emu (v3.5.4), which applies an expectation–maximization algorithm to improve taxonomic resolution and reduce false-positive assignments.

*3. PacBio Revio (full-length 16S rRNA sequencing)*

PacBio circular consensus sequencing (CCS) reads were converted from BAM to FASTQ format using bam2fastq. Quality assessment was performed using a custom Python script. For QIIME2-based analysis, reads were processed using the denoise-ccs workflow with default parameters, retaining reads with lengths between 1,200 and 1,600 bp. For the Emu-based workflow, primer sequences were removed using lima (SMRT Link), followed by quality and length filtering with chopper using the same thresholds applied to ONT data (average Phred score ≥ Q20; length 1,200–1,600 bp). Post-filtering quality checks were conducted prior to taxonomic classification.

*4. Taxonomic filtering and downstream analyses*

For all platforms and workflows, taxonomic profiles were filtered to remove reads that could not be assigned at the domain level (unassigned), as well as sequences classified as Archaea, mitochondria, or chloroplasts. Classified reads were defined as reads assigned to at least one taxonomic rank based on the reference database. The number of non-redundant classified taxa was defined as the count of unique taxonomic labels at a given taxonomic rank after excluding unassigned taxa.

**Statistical analysis**

All statistical analyses were conducted in R (version 4.5.0). The average proportion of classified reads and the number of non-redundant classified taxa were calculated for each reference database.

Comparisons of alpha diversity (observed genera) among analytical workflows across sequencing platforms were performed at the genus level due to the limited species-level resolution of short-read amplicon sequencing. Rarefaction curves based on observed genera were generated to assess sequencing depth adequacy across platforms, workflows, and reference databases. Statistical differences were assessed using the Friedman test, followed by Conover post-hoc pairwise comparisons with false discovery rate correction to account for the non-independence of observations within samples. Beta diversity differences were assessed using permutational multivariate analysis of variance based on Bray-Curtis dissimilarity with 9,999 permutations. Major genera and species were defined as those with a prevalence $\text{≥}$ 50% of samples within each sequencing platform. To assess concordance of taxonomic profiles across sequencing platforms and analytical workflows, Spearman’s rank correlation coefficients were evaluated using effective Shannon diversity at the species level. Correlation strength was interpreted using standard thresholds: weak (*r* < 0.40), moderate (0.40 $\leq$ *r* < 0.70), and strong (r $\geq$ 0.70). Statistical differences in major genera and species across workflows were evaluated using MaAsLiN3 [11], which incorporates both abundance and prevalence models. When two models showed opposing directions, the effect with the larger absolute beta coefficient was selected to represent the dominant association, thereby prioritizing the stronger signal. Statistical significance was assessed at *P* < 0.05.

**Reference**

1. Li F, Hitch TCA, Chen Y, Creevey CJ, Guan LL*.* Comparative metagenomic and metatranscriptomic analyses reveal the breed effect on the rumen microbiome and its associations with feed efficiency in beef cattle. *Microbiome* 2019;**7**:6. <https://doi.org/10.1186/s40168-019-0618-5>

2. Li F, Li C, Chen Y, Liu J, Zhang C, Irving B *et al.* Host genetics influence the rumen microbiota and heritable rumen microbial features associate with feed efficiency in cattle. *Microbiome* 2019;**7**:92. <https://doi.org/10.1186/s40168-019-0699-1>

3. Hernandez-Sanabria E, Guan LL, Goonewardene LA, Li M, Mujibi DF, Stothard P  *et al.* Correlation of particular bacterial pcr-denaturing gradient gel electrophoresis patterns with bovine ruminal fermentation parameters and feed efficiency traits. *Applied and Environ Microbiol* 2010;**76**:6338-50. <https://doi.org/10.1128/AEM.01052-10>

4. Yu Z, Morrison M. Improved extraction of pcr-quality community DNA from digesta and fecal samples. *BioTechniques* 2004;**36**:808-12. <https://doi.org/10.2144/04365ST04>

5. Kittelmann S, Seedorf H, Walters WA, Clemente JC, Knight R, Gordon JI *et al.* Simultaneous amplicon sequencing to explore co-occurrence patterns of bacterial, archaeal and eukaryotic microorganisms in rumen microbial communities. *PLoS One* 2013;**8**:e47879. <https://doi.org/10.1371/journal.pone.0047879>

6. Liao C, Rolling T, Djukovic A, Fei T, Mishra V, Liu H *et al.* Oral bacteria relative abundance in faeces increases due to gut microbiota depletion and is linked with patient outcomes. *Nat Microbiol* 2024;**9**:1555-65. <https://doi.org/10.1038/s41564-024-01680-3>

7. Hrovat K, Dutilh BE, Medema MH, Melkonian C*.* Taxonomic resolution of different 16s rrna variable regions varies strongly across plant-associated bacteria. *ISME Commun* 2024;**4** <https://doi.org/10.1093/ismeco/ycae034>

8. Comeau André M, Douglas Gavin M, Langille Morgan GI. Microbiome helper: A custom and streamlined workflow for microbiome research. *mSystems* 2017;**2**:10.1128/msystems.00127-16. <https://doi.org/10.1128/msystems.00127-16>

9. Park C, Kim SB, Choi SH, Kim S*.* Comparison of 16s rrna gene based microbial profiling using five next-generation sequencers and various primers. *Front Microbiol* 2021;**Volume 12 - 2021** <https://doi.org/10.3389/fmicb.2021.715500>

10. Lee HB, Jeong DH, Cho BC, Park JS*.* Comparative analyses of eight primer sets commonly used to target the bacterial 16s rrna gene for marine metabarcoding-based studies. *Front Mar Science* 2023;**Volume 10 - 2023** <https://doi.org/10.3389/fmars.2023.1199116>

11. Nickols WA, Kuntz T, Shen J, Maharjan S, Mallick H, Franzosa EA *et al.* Maaslin 3: Refining and extending generalized multivariable linear models for meta-omic association discovery. *Nat Methods* 2026;**23**:554-64. <https://doi.org/10.1038/s41592-025-02923-9>

**Supplementary Figure legends**

**Figure S1**. **Bioinformatic workflow of 16S rRNA sequencing and taxonomic analysis pipelines for Illumina NextSeq2000, Nanopore MinION, and Pacific Biosciences Revio platforms.**

**Figure S2. Rarefaction curves based on observed genera across sequencing depth for different reference databases under each sequencing platform using rumen samples from 23 Kinsella composite hybrid steers.** A. Illumina NextSep2000, B. Nanopore MinION analyzed using EPI2ME, C. Nanopore MinION analyzed using Emu, D. PacBio Revio analyzed using QIIME2, and E. PacBio Revio analyzed using Emu.

**Figure S3. Box plots showing difference in observed genera among reference databases across sequencing platforms.** SILVA, SILVA 138.2; SilHun, SILVA 138.2 with Hungate1000 collection; NCBI, NCBI RefSeq 16S rRNA; GG2, Greengenes2 (2024.9) and GTDB, GTDB (10-RS226).

**Figure S4. Beta diversity of rumen bacterial communities across sequencing platforms and reference databases.** Principal coordinates analysis based on Bray-Curtis dissimilarity showing the clustering of samples across sequencing platforms under different reference databases. SILVA, SILVA 138.2; SilHun, SILVA 138.2 with Hungate1000 collection; NCBI, NCBI RefSeq 16S rRNA; GG2, Greengenes2 (2024.9) and GTDB, GTDB (10-RS226).

**Figure S5. Taxonomic distribution of major genera across sequencing platforms under different reference databases.** (A) SILVA 138.2, (B) SILVA 138.2 with Hungate1000 collection, (C) NCBI RefSeq16S rRNA, and (D) Greengenes2 (2024.9) databases. Stacked bar plots represent the relative abundance (%) of major genera across sequencing platforms. Major genera were defined as those with a prevalence $\text{≥}$ 50% of samples within each sequencing platform and a relative abundance $\text{≥}$ 2.0% in at least one platform. “Others” indicates the combined relative abundance of taxa not classified as major genera. Numbers within bars indicate rank and relative abundance (%) of the top five genera within each platform.

**Figure S6.** **Comparison of species-level diversity and major species across sequencing platforms in 23 Kinsella composite hybrid steers and Angus bulls based on the GTDB reference database.** (A) Effective number of species (Shannon diversity) across platforms. Statistical significance was assessed using the Friedman test followed by Conover post-hoc comparisons with FDR correction. Different letters indicate statistically significant differences among platforms within each cohort. (B) Number of major species retained after filtering based on relative abundance and prevalence thresholds. Major species were defined as those with a prevalence ≥ 50% of samples within each sequencing platform and a relative abundance ≥ 0.05% in at least one platform. Bars represent the total number of major species detected for each platform. Counts represent the number of species meeting these criteria within each platform–database combination.

**Figure S7. Pairwise Spearman correlation of z-score-normalized effective Shannon diversity at the species level across sequencing platforms based on the GTDB classification.** Each point represents an individual sample, colored by breed (KC Steers, blue; AN Bulls, red). Linear regression lines with 95% confidence intervals are shown for visualization. KC, Kinsella composite hybrid; AN, Angus.

**Figure S8. Genus-level taxonomic distribution and differential abundance of major genera across sequencing platforms under GTDB classification in two independent rumen datasets.** (A) Stacked bar plots showing the relative abundance (%) of major genera across sequencing platforms. Major genera were defined as those with a prevalence $\text{≥}$ 50% of samples within each sequencing platform and a relative abundance $\text{≥}$ 2.0% in at least one platform. “Others” indicates the combined relative abundance of taxa not classified as major genera. Numbers within bars indicate rank and relative abundance (%) of the top five genera within each platform. (B) Pairwise differential abundance and prevalence analysis of major genera across sequencing platforms based on MaAsLiN3. Tiles are colored according to beta coefficients, and labels indicate the dominant model type: A, abundance; P, prevalence; AP, both models with concordant direction. When abundance and prevalence models showed opposite directions, the model with the larger absolute beta coefficient was selected.

**Supplementary Table legends**

**Table S1. Summary of raw read quality statistics for Illumina NextSeq 2000 16S rRNA amplicon sequencing from 23 Kinsella composite hybrid steers.**

**Table S2. Summary of raw read quality statistics for Nanopore MinION full-length 16S rRNA sequencing from 23 Kinsella composite hybrid steers and 24 Angus bulls.**

**Table S3. Summary of raw read quality statistics for PacBio Revio full-length 16S rRNA sequencing from 23 Kinsella composite hybrid steers and 24 Angus bulls.**

**Table S4. Relative abundance of *Prevotella* species based on GTDB (10-RS226) database across sequencing platforms from 23 Kinsella composite hybrid steers.**

**Table S5. Relative abundance of *Prevotella* species based on GTDB (10-RS226) database across sequencing platforms from 24 Angus bulls.**
