# Supplementary figures and images for: PacBio full-length 16S rRNA gene sequencing processed with Emu and GTDB provides the highest taxonomic resolution for rumen bacteriome profiling
Source: ISME Commun. 2026 May 29;6(1):ycag148. doi: 10.1093/ismeco/ycag148 (PMC13289738; doi:10.1093/ismeco/ycag148)

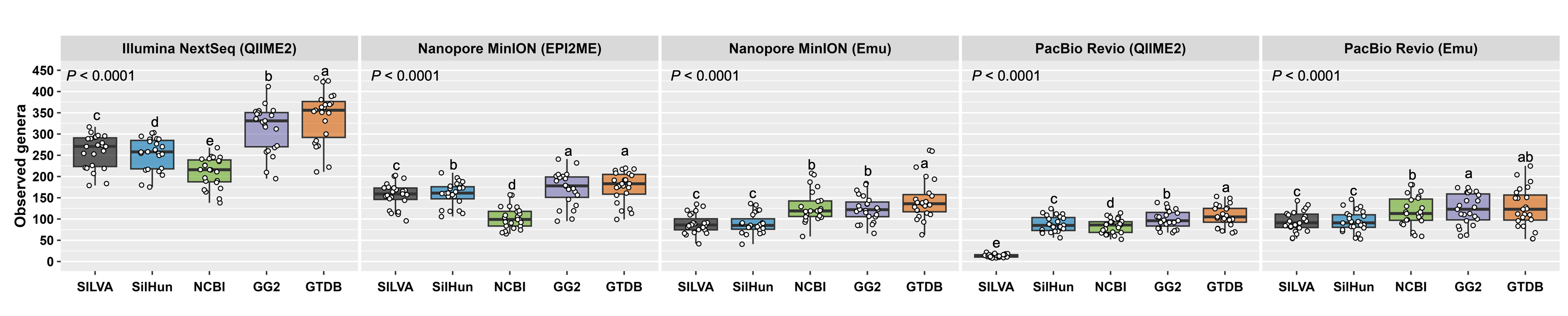

Supplement: Supplementary_material_ycag148 [file supplementary_material_ycag148.zip › Fig_S3.png]

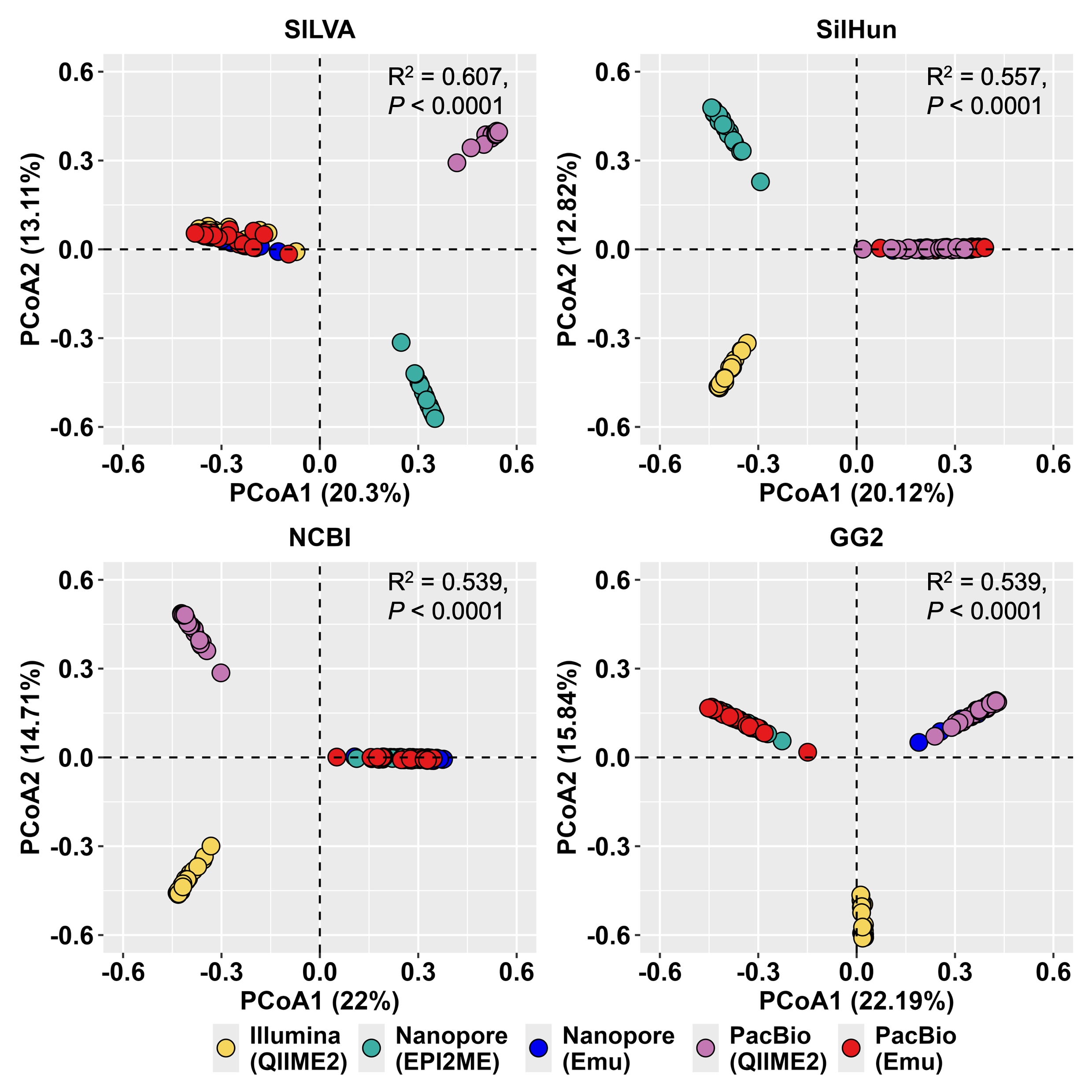

Supplement: Supplementary_material_ycag148 [file supplementary_material_ycag148.zip › Fig_S4.png]

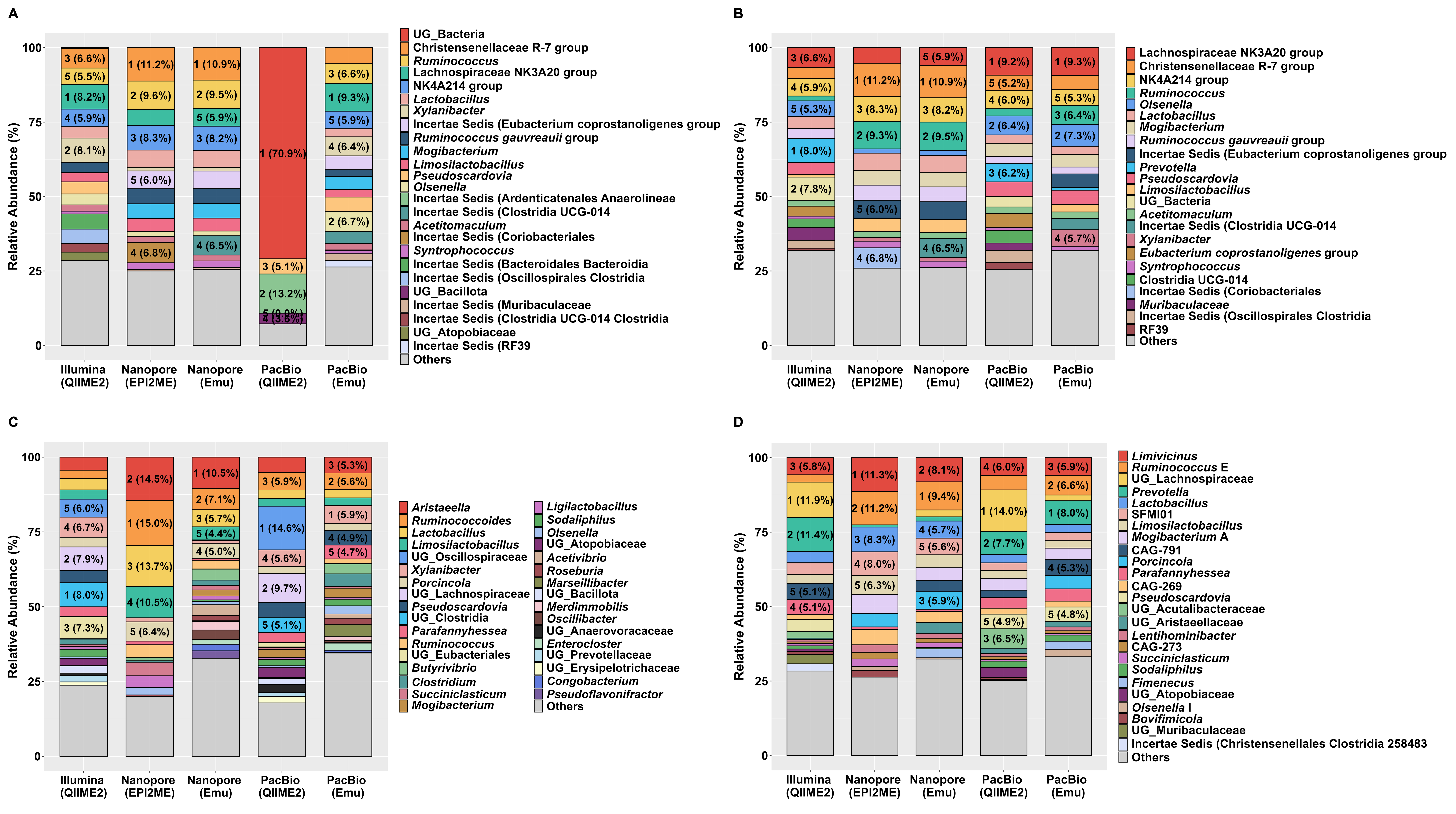

Supplement: Supplementary_material_ycag148 [file supplementary_material_ycag148.zip › Fig_S5.png]

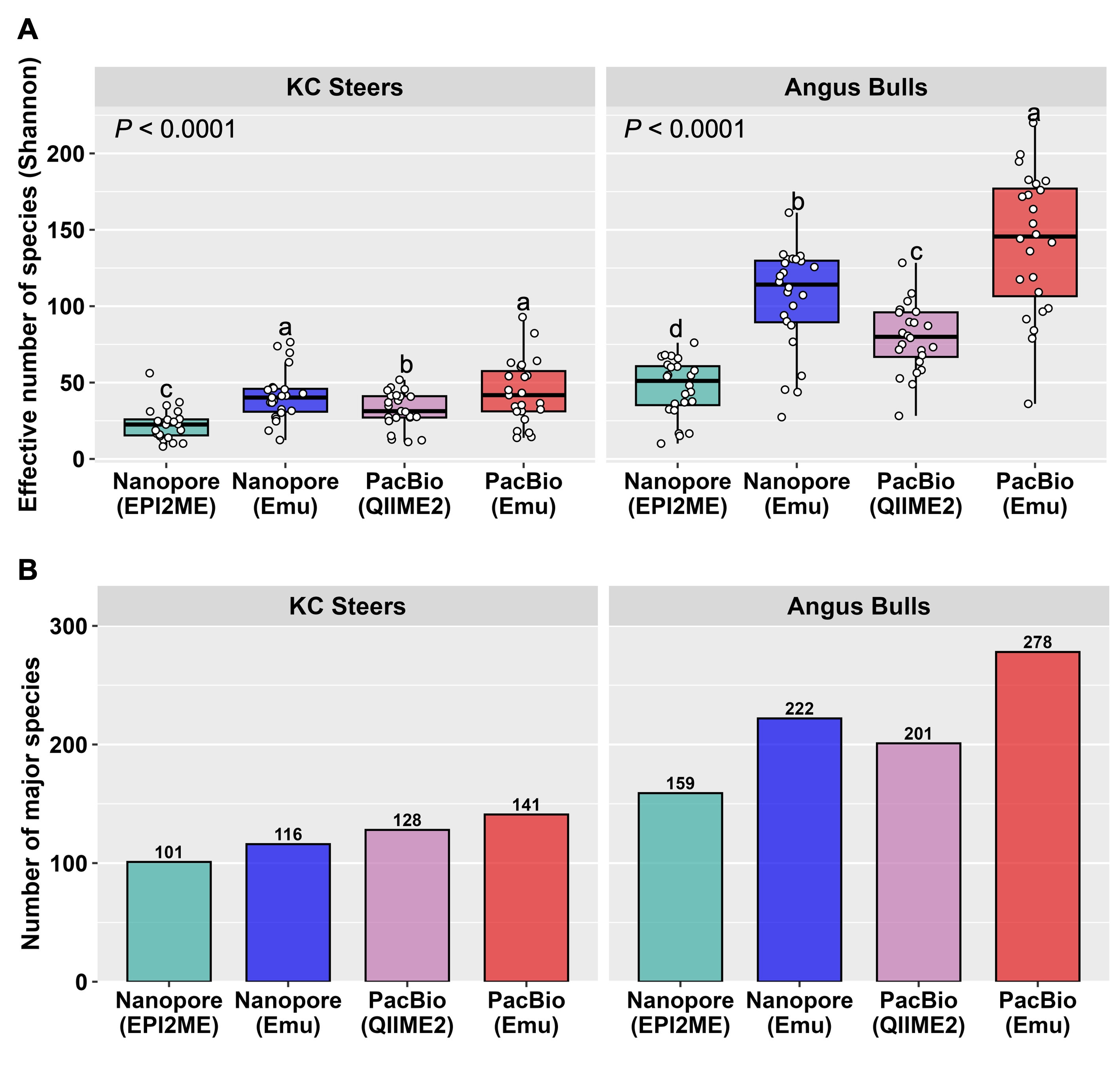

Supplement: Supplementary_material_ycag148 [file supplementary_material_ycag148.zip › Fig_S6.png]

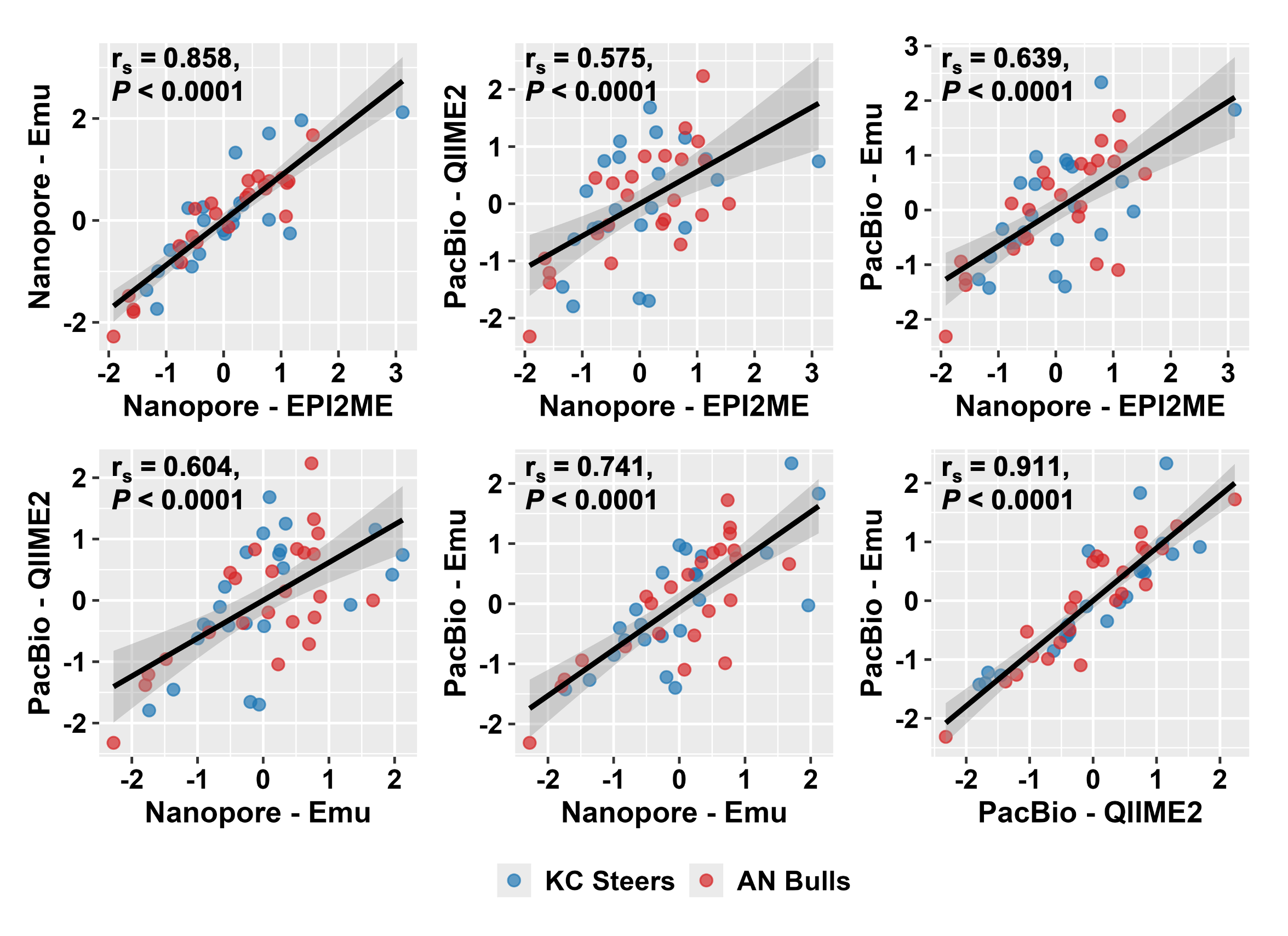

Supplement: Supplementary_material_ycag148 [file supplementary_material_ycag148.zip › Fig_S7.png]

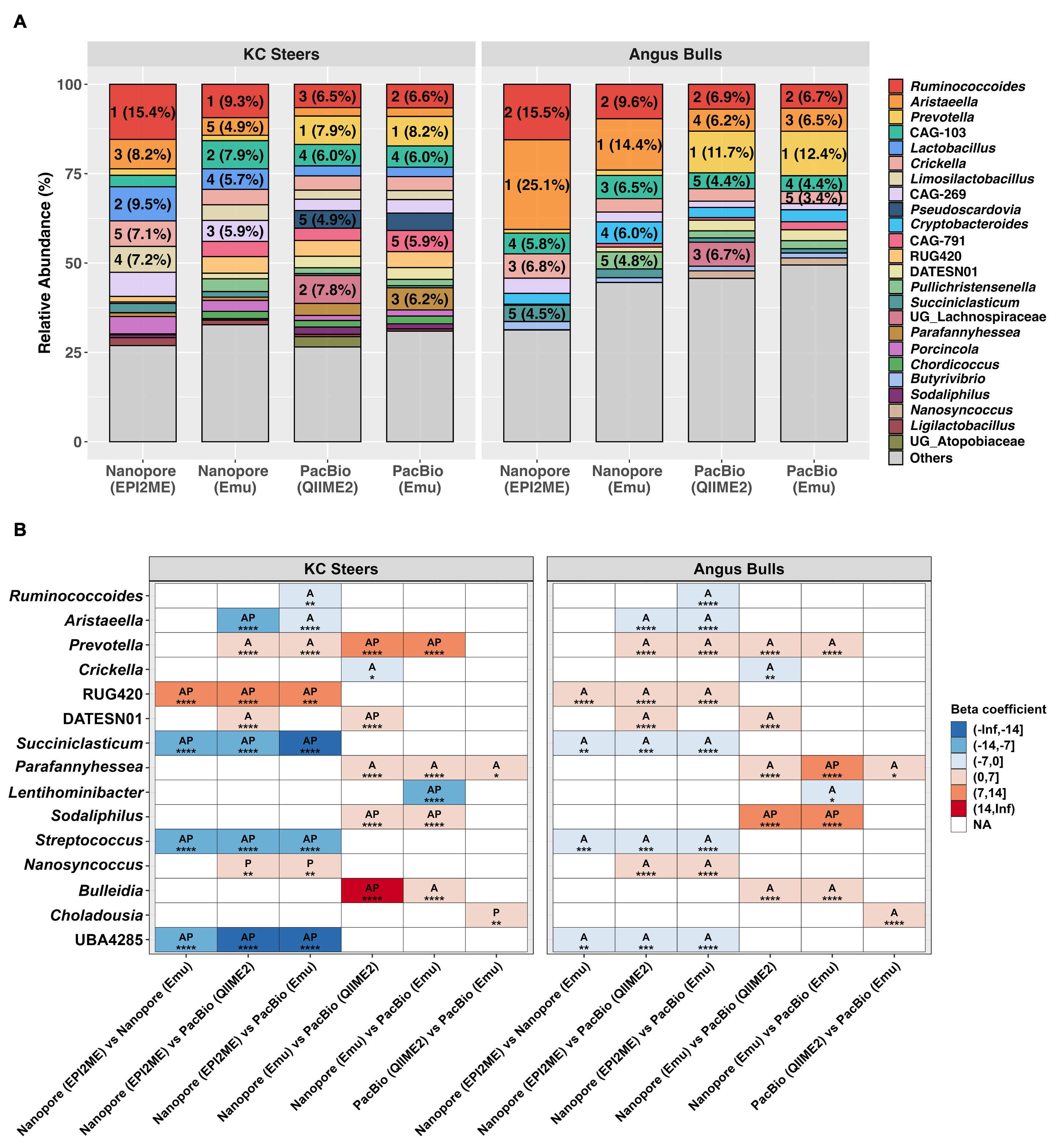

Supplement: Supplementary_material_ycag148 [file supplementary_material_ycag148.zip › Fig_S8.png]

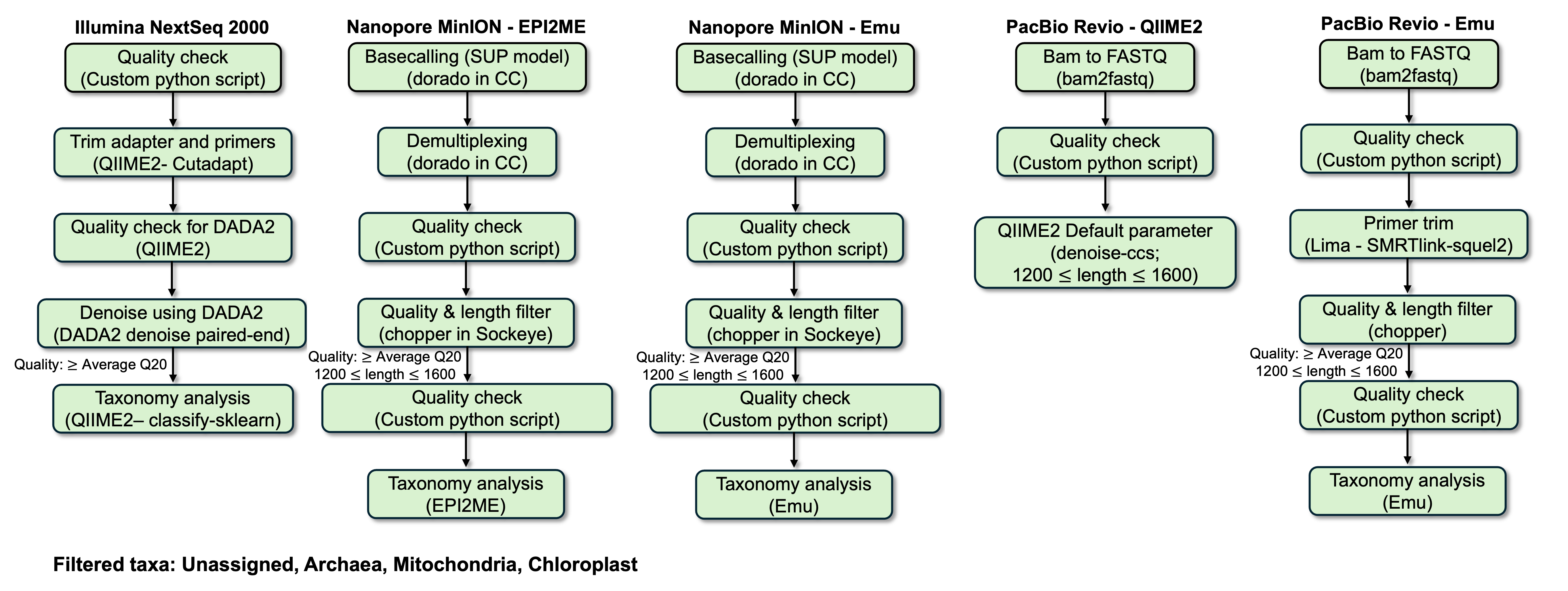

Supplement: Supplementary_material_ycag148 [file supplementary_material_ycag148.zip › Fig_S1.png]

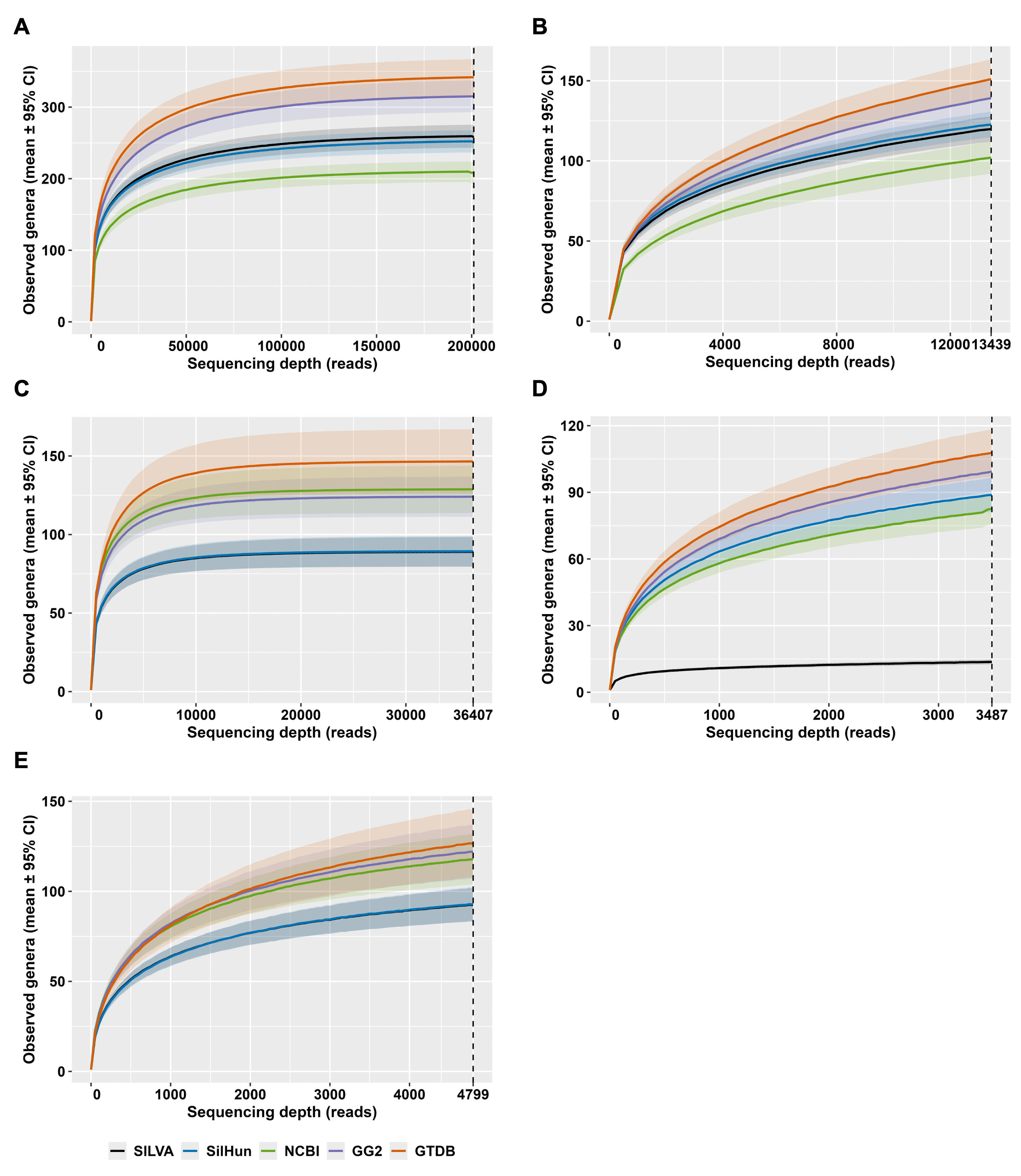

Supplement: Supplementary_material_ycag148 [file supplementary_material_ycag148.zip › Fig_S2.png]
